# A most important gift: the critical role of postmortem brain tissue in autism science

**DOI:** 10.3389/fneur.2024.1486227

**Published:** 2024-12-11

**Authors:** Marvin R. Natowicz, Margaret L. Bauman, Stephen M. Edelson

**Affiliations:** ^1^Pathology and Laboratory Medicine, Genomic Medicine, Neurological and Pediatrics Institutes, Cleveland Clinic, Cleveland, OH, United States; ^2^Cleveland Clinic Lerner College of Medicine of Case Western Reserve University, Cleveland, OH, United States; ^3^Department of Anatomy and Neurobiology, Boston University School of Medicine, Boston, MA, United States; ^4^Autism Research Institute, San Diego, CA, United States

**Keywords:** autism, autopsy, brain, brain donation, neuropathology, postmortem

Autism spectrum disorder (hereafter, autism or ASD) is a common and complex neurodevelopmental condition that affects approximately 1% of the population worldwide (1, 2). As currently defined, the core features of autism include persistent deficits in social communication and social interaction in multiple settings, accompanied by restricted, repetitive patterns of behavior, interests, or activities (3). There is, however, substantial variability in the clinical presentation and natural history of affected individuals (1, 2, 4). In the majority of instances, the etiological basis for an individual's autism is not determined, the underlying biological mechanisms are poorly understood, and clinical care is often suboptimal (1, 2, 4). Gaining a more complete understanding of the processes affecting brain biology in autistic persons is important for addressing one of the major global public health challenges of our time: the development of effective care for autistic persons (4). This article presents the case for ASD postmortem brain research and the need for ASD brain donations.

## Why is studying human brain tissue important in autism science?

There are many established and useful approaches to learning about the causes and associated neurobiological mechanisms related to ASD, many of which do not require an analysis of human brain tissue. Some of these include various types of epidemiological investigations, clinical cohort analyses, studies of genetics or biochemistry of peripheral human tissues such as blood or urine, and neuroimaging studies of children and adults. Some of the central lessons learned from these types of studies include the knowledge that ASD is a common and both etiologically and clinically highly heterogeneous condition, that autism has high heritability, and that several neuroradiological findings are more commonly noted in autistic persons (1, 2). In addition, multiple investigations have shown that many rare, highly penetrant genetic variants associated with autism involve such biological processes as chromatin remodeling and synapse organization and function (5). It is therefore important to ask: why is the procurement of autistic brain tissue of such importance for the advancement of knowledge about autism?

While the majority of persons with autism have one or more co-occurring medical conditions that involve tissues outside of the brain (1, 2, 4), the core organ system related to ASD is the brain. It is crucial, then, to understand how, where, and when the brain is affected. A major challenge in accomplishing this goal is that many important characteristics and processes of brain biology can only be understood through the direct investigation of brain tissue obtained from individuals with ASD.

## What types of studies can be carried out using human brain tissue, and what types of information can be learned from these studies?

Many different types of studies can be carried out using human brain tissue, all of which have the potential to provide valuable information about ASD. Some of the major types of studies that have been carried out include histological analyses, gene expression studies, and various types of biochemical analyses including measurements of proteins, lipids, and other metabolites. Each of the major types of studies that have been carried out on the postmortem ASD brain is briefly reviewed below.

### Histological and histochemical analyses

Analyzing brain tissue from individuals with ASD offers the opportunity to examine ASD-related biological changes associated with neuronal and glial cell organization, synaptic connectivity, and neurotransmitter systems. By studying brain tissue, researchers can observe how neurons and other cell types are arranged and connected, revealing potential abnormalities in the neural networks those are critical for communication, behavior, and cognition. Analysis of neurotransmitter systems in brain tissue can provide insight into potential chemical imbalances that may contribute to the symptoms of autism, thus offering possible targets for pharmacological intervention. In addition, research involving the study of brain tissue can also provide insights into brain plasticity, the brain's ability to change in response to experiences. This may be especially relevant for individuals with ASD, where an understanding of potential changes that may be part of development and/or in response to medical and therapeutic interventions may provide insight into the brain processes in ASD and suggest specific therapeutic approaches.

Histological/histochemical analyses of postmortem ASD brain have yielded important, but sometimes varied or inconsistent results. These differences are likely attributable to variations in the brain regions evaluated in different studies, differences in methodological approaches, differences in underlying etiology, and variations in the age and sex of donors, as well as differences in co-occurring conditions, treatments, and causes of death (6, 7). Histological abnormalities have been noted in different regions of the cerebral cortex (e.g., the prefrontal and frontal cortex, fusiform gyrus, frontoinsular and cingulate cortex, and hippocampus) (6, 7). Non-cortical regions that are consistently altered in ASD include the amygdala and cerebellum (6, 7). Abnormalities that have been observed include differences in neuronal size, number, density and the disposition of their axons and dendritic spines, as well as histological changes in non-neuronal cells and myelination (6, 7). A substantial subset of ASD brains shows evidence of neuroinflammation (8). Many of the observed histological differences relate to brain regions involved in motor control, cognition, and emotional regulation.

### Gene expression analyses

Additional information regarding the biology of autism that has been obtained through analyses of postmortem brain tissue has included studies of ASD gene expression changes and the upregulation or downregulation of different genes in the autistic brain compared to the control brain tissue. The regulation of gene expression is complex and requires the normal function of different steps from the initial transcription of the information present in DNA to the final production and localization of the end product RNAs, messenger RNAs, and non-coding RNAs. Dysregulation of gene expression can be due to an inherited or *de novo* genetic variant or consequent to a non-genetic process that alters one or more steps of the gene expression process. Non-genetic occurrences that can impact gene expression in the brain include changes in the prenatal or postnatal brain cellular environment due to altered levels or new exposures to hormones, nutritional changes, infections, toxins, and other exposures. These effects are sometimes mediated through epigenetic processes. Regardless of the cause of gene dysregulation, changes in gene expression can be identified by measurement of the levels of many mRNA and non-coding RNA species (transcriptomic analysis), and in cases of epigenetic dysregulation, specific epigenetic signatures, such as particular gene methylation patterns, can be identified. Many changes in ASD gene expression patterns have been identified, with some convergence observed of dysregulated genes and pathways, including in the ASD brain (9–11). The majority of ASD brain gene expression studies have involved analyses of tissue samples consisting of numerous brain cells, sometimes referred to as bulk gene expression studies, an approach that lacks cell-specific resolution. The use of newer methods to explore single-brain cells or single-cell nuclei has enabled gene expression studies to be carried out on specific cell types from different regions of the postmortem brain. These powerful approaches, in turn, have disclosed cell-specific information that has confirmed and extended the gene expression analyses of larger brain tissue samples (12–18). Information derived from detailed gene expression, other genetic, epigenetic, and biochemical analyses of single cells, when integrated with information from cells across all brain regions and across ages and phenotypes, will facilitate a markedly increased understanding of the underlying brain biology of ASD (19, 20). Like the histological studies mentioned above, research on changes in ASD brain gene expression relies on investigations using donated postmortem ASD brain tissue.

### Biochemical analyses

In addition to histological and gene expression studies of ASD postmortem brain tissue, there have been different types of biochemical studies ranging from analyses of specific metabolites or families of metabolites to analyses of one or more proteins in autistic brains. It is beyond the scope of this article to describe the large number of studies that involve analyses of single or several metabolites or proteins whereas to date there are still only a few large-scale metabolic (metabolomic) (21, 22) or large-scale protein (proteomic) (23–25) analyses of the ASD brain. The strength of this level of experimental analysis of brain tissue is that the molecules of study—such as proteins or metabolites—are the “agents” of altered gene expression, making them “closer” to the phenotype of altered brain function observed in autism.

The diverse investigations—histological, gene expression, and biochemical—of autism using autistic brains have yielded important insights regarding the biology of the autistic brain, the majority of which would not have been achieved without the availability of ASD postmortem brain specimens. An early study of ASD brain is a historically important example of this. The first substantive investigation of postmortem ASD brain, histological analysis of a single adult brain published in 1985, revealed distinctive regional histological abnormalities and, importantly, surmised that, at least in that individual, these brain abnormalities had to have occurred during prenatal development (26). This, in turn, fundamentally challenged the then commonly held hypothesis of autism as a consequence of poor parenting.

## What are the major challenges for scientists who need brain tissue for ASD research?

Researchers who use ASD brains for their scientific work are faced with important challenges. One significant challenge is the limited number of ASD brains that are available for investigative work, along with related issues such as poorly characterized specimens and the compromised quality of some tissues, which can hinder investigative efforts. Another common limitation is the often incomplete clinical and other ancillary information about the individuals who donate their brains. This lack of detailed data can limit the effectiveness and, in some cases, the use of donated brain tissue for important research projects.

Currently, the NIH NeuroBioBank network (27) has brain specimens from >3,200 subjects with Alzheimer's disease, >400 subjects with amyotrophic lateral sclerosis disease, >700 subjects with Huntington's disease, >1,600 subjects with multiple sclerosis, >1,000 subjects with schizophrenia, and >1,100 subjects with Parkinson's disease, but there are only 106 brain specimens from subjects with ASD.^1^ Similarly, the total number of fixed or frozen brain tissues available through the Autism BrainNet network (28) includes 204 specimens, although the number of individual subjects is < 204, as the brain tissue from some subjects was divided into both fixed and frozen hemispheres.^2^ While each of these neurological disorders has much personal and societal clinical significance and requires continued research attention, the disparity in the number of ASD postmortem brains that are available for research compared to the number of banked brains from many other conditions is striking, especially when the number of ASD brains is considered relative to the number of available brains from other conditions with approximately similar or even lower population prevalence (29, 30). In 2021, for example, the age-standardized population prevalence of ASD was 783.3 per 100,000, and the population prevalence of Alzheimer's disease and other dementias, Parkinson's disease, multiple sclerosis, and motor neurone disease were 694/100,000, 138.6/100,000, 22.2/100,000, and 3.3/100,000 (30) respectively. However, as noted above, these neurological conditions have considerably greater numbers of postmortem brains available for research in the NIH NeuroBioBank.

## What is the process for donating to a brain tissue bank?

Parents and family members should discuss the possibility of donating their loved one's brain to a tissue bank for research use. This decision can be difficult and may be influenced by personal, religious, and/or cultural beliefs. If the decision to donate the loved one's brain to a tissue bank for research purposes has been made in advance of the individual's death, the family may contact a brain bank in advance. Contact with the brain bank before a donor's death is important, when possible. The passing of a loved one is usually a stressful time, and having to locate and contact a tissue bank and then provide the necessary information can make the situation even more challenging.

Autism BrainNet (www.AutismBrainNet.org) consists of a network of three U.S. sites and two international partnerships. There is a 24-h hotline number (+1-877-333-0999) that a family member can call to initiate the donation process. The answering staff will then walk the family through the process of brain donation. This process involves determining if the donor meets the inclusion and exclusion criteria and, if so, completing a consent process to authorize the research use of the brain and the acquisition of medical records. A clinical director then facilitates the process of obtaining the brain donation in the shortest time by working with a local coroner or regional collaborating brain bank (28).

In some instances, the demise of a loved one is unanticipated, sometimes due to an accident or a new illness, and there may not have been prior consideration of the possibility of a brain donation. In this situation, donation of the brain can still occur. The family can contact the 24-h hotline above and the donation process can proceed as described. In addition, pathologists, coroners, and medical examiners can also contact Autism BrainNet for assistance with ASD brain donation in instances where potential donors are first encountered at autopsy (31).

There are other high-quality brain banks available for families considering brain donation. The National Institutes of Health NeuroBioBank, accessible through the Brain Donor Project (www.BrainDonorProject.org; 513-393-7878), oversees a network of brain banks across six U.S. locations. These sites collect brain tissue from individuals with various neurological, neuropsychiatric, and neurodevelopmental disorders. Participating institutions include the University of Maryland Brain and Tissue Bank (800-847-1539), the University of Miami Brain Endowment Bank (800-UM-BRAIN), the Brain Tissue Donation Program at the University of Pittsburgh (513-393-7878), the Harvard Brain Tissue Resource Center (800-BRAIN-Bank), the Mount Sinai Brain Bank (212-807-5541), and the Human Brain and Spinal Fluid Resource Center (310-268-3330).

Both the Autism BrainNet and the NIH NeuroBioBank networks encourage pre-registration and have user-friendly web-based and phone-based systems to accomplish this. The Autism BrainNet network is focused on advancing autism brain research; the NIH NeuroBioBank network is a resource for research on many types of brain disorders. Both networks, along with other brain banks (32, 33),^3^ accept donations of neurotypical brains. The importance of the latter also needs special mention; donated neurotypical brains serve as critically important controls in scientific investigations (34). High-quality postmortem-based brain research requires close matching of cases and controls with respect to all key parameters, typically including matching on age, sex, postmortem interval, and region of the brain studied. Additionally, there often are other crucial inclusion/exclusion criteria such as the absence of certain conditions (e.g., brain trauma, brain hemorrhage, primary or metastatic brain tumor, recent stroke or seizure, and use of certain medications). Having a large pool of control brains increases the likelihood that control brain samples are not exhausted and that cases and controls can be appropriately matched on all essential variables.

Overall, the donation of a brain for research purposes is a complex process, from the procurement of the brain to its ultimate use in research investigations. Each step of the process requires forethought and appropriate implementation, ranging from the respectful interaction with the family and collection of needed medical information to time-sensitive logistical issues regarding the timely handling and processing of the brain, to the dispensing of tissue for needed investigation.

Finally, we recognize that not all families or pathologists interested in participating in ASD brain donation may have close geographic proximity to a center that specializes in this effort. The brain banks of the NIH are not able to accept brains from outside of the United States. Autism BrainNet has two collaborating centers outside of the United States: the Douglas-Bell Canada Brain Bank (douglasbrainbank.ca; 514-761-6131ext.3496), a McGill University affiliate, and the Oxford Brain Bank in the United Kingdom (brainbank@ndcn.ox.ac.uk). For those situations where a brain donation from an ASD donor is desired and the deceased individual is not in a location with timely access to one of the described networks, the family or pathologist may consider an online search for brain donation programs in that country or region.

## Conclusion

The collection and analysis of human brain tissue are indispensable for advancing the understanding of ASD. Current technologies are unable to study the living human brain at the level of resolution needed to understand the cellular and molecular alterations present in the ASD brain. Thus, much vital information about the biological basis of autism can only be learned through investigation of the postmortem brain. Insights gained from studying postmortem brain tissues result in an improved understanding of the cellular and molecular basis of the neurobiology of autism. This, in turn, will help enable the development of innovative therapeutic approaches that hold the promise of transforming lives.

## Footnotes

^1^https://neurobiobank.nih.gov (accessed August 19, 2024).

^2^https://www.autismbrainnet.org (accessed August 19, 2024).

^3^https://brainfoundation.org.au/support-us/donate-your-brain/ (accessed September 23, 2024).
